# Comparative Assessment of Phenolic Content and *in Vitro* Antioxidant Capacity in the Pulp and Peel of Mango Cultivars

**DOI:** 10.3390/ijms160613507

**Published:** 2015-06-12

**Authors:** Arshad Mehmood Abbasi, Xinbo Guo, Xiong Fu, Lin Zhou, Youngsheng Chen, Yong Zhu, Huaifeng Yan, Rui Hai Liu

**Affiliations:** 1 School of Light Industry and Food Sciences, South China University of Technology, Guangzhou 510641, China; E-Mails: amabbasi@ciit.net.pk (A.M.A.); roger1887@163.com (X.F.); zhoulin@gdpu.edu.cn (L.Z.); chysh11@126.com (Y.C.); zhuyonghappycool@163.com (Y.Z.); jinianjin@outlook.com (H.Y.); 2 Department of Environmental Sciences, COMSATS Institute of Information Technology, Abbottabad 22060, Pakistan; 3 Department of Food Science, Cornell University, Ithaca, NY 14850-2824, USA; 4 Guangdong Province Key Laboratory for Biotechnology Drug Candidates, School of Biosciences and Biopharmaceutics, Guangdong Pharmaceutical University, Guangzhou 510006, China

**Keywords:** mango, phenolics, flavonoids, anthocyanins, phenolic acids, ORAC, hydro-PSC

## Abstract

Mango (*Mangifera indica* L.), also called “the king of fruits”, is one of the most popular fruits in tropical regions. Pulp and peel samples of mango cultivars were analyzed to estimate total phenolic, total flavonoid and total anthocyanin contents. Phenolic acids, hydrophilic peroxyl radical scavenging capacity (hydro-PSC) and oxygen radical scavenging capacity (ORAC) *in vitro* were also determined. Total phenolics and flavonoid contents were found maximum in the peel of Xiao Tainang and Da Tainang cultivars, respectively, whereas Xiao Tainang also exhibited significant antioxidant capacity. Noteworthy, concentrations of gallic acid, protocatechuic acid, ferulic acid, chlorogenic acid and caffeic acids at 79.15, 64.33, 33.75, 27.19 and 13.62 mg/100 g fresh weight (FW) were quantified for Da Tainang, Xiao Tainang and of Jidan cultivars, respectively. Comparatively, a higher level of phenolics and significant antioxidant capacity in mango peel indicated that it might be useful as a functional food and value-added ingredient to promote human health.

## 1. Introduction

Humans have relied on nature throughout the ages for their basic needs of food and health. At present, there is an increasing interest in exploring new sources of plant bio-actives for applications in both the food and pharmaceutical industries [1]. Fruits and vegetables are the most important food sources, which supply essential nutrients and also contain an array of phytochemicals, such as phenolics and flavonoids, to maintain good health [2]. Fruits provide an opportunity for local growers to access the specialized markets where consumers show a preference for exotic characteristics and the presence of nutrients in food, capable of preventing degenerative diseases [3]. A number of reactive oxygen species (ROS), including superoxide anion, hydroxyl and hydrogen peroxide radicals, are produced in the human body by numerous enzymatic systems through oxygen consumption. These reactive oxygen species cause cancer, cardiovascular diseases, aging and neurodegenerative disorders [4]. The ingestion of fruits and vegetables has been connected with a distinguished health-protecting factor against diseases caused by oxidative stress [5,6].

Health benefits of fruits and vegetables have been attributed partly to the compounds having antioxidant capacity and an ability to overcome oxidative stress by neutralizing the overproduction of oxidant species [7,8]. It has been reported that the additive and synergistic effects provided by the complex mixture of phytochemicals present in fruits and vegetables cannot be achieved through micronutrient supplements [9]. Polyphenolic compounds, including phenolic acids, xanthones, gallotannins, carotenoids and vitamins (E and C), are important anti-radical, anti-mutagenic and anti-carcinogen agents [10,11]. They reduce the risk of chronic diseases, because of their safety, effectiveness and the presence of hydroxyl groups, which enable these compounds to have more diverse biological activities [12]. Phenolics antioxidants, such as hydroxyl benzoic acid, and their derivatives are potent free radical scavengers of singlet oxygen possibly concerning DNA damage and tumor promotion [13]. Phenolic acids are predominant compounds in the pulp of mango [14]. Consumption of ripened mango is better, as it contains a high content of phenolic acids, which play a significant role in quenching and neutralizing the free radicals to improve consumers’ health [15,16]. Gallic acid is one the important anticancer agents, particularly against human prostate cancer cells *in vitro* and *in vivo* [17]. Ferulic acid is an important phenolic compound in fruits and vegetables, which is generated as a result of phenylalanine and tyrosine metabolism [18]. By virtue of effectively scavenging harmful radicals and suppressing radiation-induced oxidative reactions, ferulic acid serves as an important antioxidant, protects the body against different inflammatory diseases and is essential in preserving the physiological integrity of cells exposed to both air and impinging UV radiation [19]. *In vitro* and *in vivo* studies have revealed that chlorogenic acid is one of the most abundant polyphenols in the human diet, which exhibits significant anti-edematogenic, anti-nociceptive, antioxidant and anti-carcinogenic activities [20]. It has been reported that protocatechuic acid showed anti-proliferative activity against HL-60 cells by inducing apoptosis and is associated with the phosphorylation and suppression of Bcl-2 protein [21]. Caffeic acid has been proven as an inhibitor of hypertension and cardiotoxicity in rats by enhanced blood pressure, cardiac injury markers, restoration of the oxidant/antioxidant status, as well as decreasing histopathological changes [22].

*Mangifera indica* L. (mango), “the king of fruits” belonging to the family Anacardiaceae, is one of the most popular fruits in tropical regions. Mango has been cultivated for 4000 years and ranks only second to pineapple in quantity and value among internationally-traded tropical fruits. In Mainland China, mango was first introduced from India in 645 A.D. by Tang Xuangzang (Tang Dynasty), and its commercial cultivation was started in the 1980s. Now, China has become the seventh mango cultivation country in the world, with annual production of about 1,061,800 tones on 133,100 hectares [23]. Mango is considered as a good source of dietary compounds, such as ascorbic acid, phenolic compounds and carotenoids [15,24,25], which are beneficial to health due to their antioxidant capacity [26,27]. The pulp of mango is effective for leukemia, prostate, breast and colon cancers *in vitro*. Peels are the major by-products of different fruits and are good sources of phytochemicals and bioactive compounds [28,29,30]. Mango peel, which comprises 15%–20% of the fruit, is an edible tissue and a major by-product of the mango processing industry. Peel of unripe mango is used in making chutney and pickle, while that of the ripe fruit, due to its leathery nature, is not so satisfactory in taste, therefore being generally removed and discarded. In the food processing industry, mango peel is removed for technological and sensory advantages and usually ends up as a waste by-product [31]. Mango peel has been found to be a good source of polyphenols, carotenoids, dietary fiber, vitamin E and vitamin C [28,31], and it showed significant antioxidant properties [32,33].

Total phenolics, vitamin C and antioxidant activity have been reported in the fruit of mango varieties [15,24,25]. However, comparative assessment of total phenolic content, phenolic acids and *in vitro* antioxidant capacity in the pulp and peel of mango cultivars predominantly cultivated in China has rarely been reported before. In this context, the present study was designed to evaluate and correlate phenolic content and antioxidant activity and to assess the disparity in phenolic compounds and antioxidant capacity in the pulp and peel of nine cultivars of mango.

## 2. Results and Discussion

### 2.1. Moisture Content

Moisture levels as the percentage of moisture content determined in the pulp and peel of mango cultivars are given in Table 1. Overall, pulp samples contain higher moisture content than peel. Interestingly, maximum moisture content was determined in the pulp and peel of the Kaite cultivar at 89.47% and 87.04%, respectively. The Da Tainang cultivar showed the lowest level of moisture at 81.68 for pulp and 77.57 for peel sample. In the present study, the percentage of moisture content of the peel samples was higher than reported previously for Raspuri and Badami mango varieties (65%–75%) from India [32], which might be due to genetic variation and climatic conditions.

**Table 1:** Descriptions of the mango cultivars and the percentage of moisture content.

Variety	Abbreviation		Color	% Moisture Content	
	Pulp	Peel		Pulp	Peel
Luzon mango (Lvsong)	F1	P1	Greenish yellow	85.13 ^ab^ ± 0.70	82.21 ^c^ ± 0.53
Narcissus mango (Shuixian)	F2	P2	Yellow	80.69 ^c^ ± 0.92	79.18 ^e^ ± 0.09
Royal mango (Guifei)	F3	P3	Yellowish red	88.53 ^a^ ± 0.32	83.49 ^b^ ± 0.55
Big Tainong mango (Da Tainang)	F4	P4	Yellow	81.68 ^bc^ ± 0.55	77.57 ^f^ ± 0.31
Keitt mango (Kaite)	F5	P5	Green	89.47 ^a^ ± 4.65	87.04 ^a^ ± 0.50
Australian mango (Aozhou)	F6	P6	Reddish yellow	85.89 ^a^ ± 0.21	83.90 ^b^ ± 0.13
Thai mango (Xiangya)	F7	P7	Green	86.01 ^a^ ± 0.38	81.66 ^cd^ ± 0.71
Small Tainong mango (Xiao Tainang)	F8	P8	Yellow	88.80 ^a^ ± 0.10	81.01 ^d^ ± 0.42
Egg mango (Jidan)	F9	P9	Yellow greenish	87.87 ^a^ ± 0.13	84.25 ^b^ ± 0.44

Values are the means of three replicates ± SD. Different letters (a–f) within the columns indicate significant difference at *p* < 0.05.

### 2.2. Total Phenolic Content

Phenolics are among the major contributors that are accountable for antioxidant properties in fruits, vegetables, whole grains and other plant-based materials [34]. Although total phenolic compounds in the mango pulp have been reported before, to our knowledge, the phenolic composition in the peel of Chinese cultivars is estimated for the first time here. The measured levels of total phenolic content (TPC) in the pulp and peel samples of mango cultivars are presented in Table 2, which indicated that peel samples contained high phenolic content compared to pulp. Our results are consistent with a previous report [35] that peel always contains more phenolic contents than pulp at any stage of mango fruit.

Peel is an important by-product of mango processing and is a good source of high-quality pectin and polyphenols [36]. On the whole, the peel and pulp of the Xiao Tainang cultivar exhibited a higher concentration of total phenolics among all of the studied samples. In peel samples, TPC ranged from 462.2–4071 mg gallic acid equivalent (GAE)/100 g fresh weight (FW). The Lvsong cultivar contained the lowest level of total phenolics, while in the peel of the Xiao Tainang variety, the TPC level was highest with a significant difference (*p* < 0.05). It was noted that the total phenolic contents reported in the peel of Pica mango from Chile [24] and the Ataulfo variety from Mexico [37] were slightly higher than the present results. Differences in the cultivars, their origins and genetic variation might result in the inconsistency among the findings [38].

In the pulp samples, the maximum concentration of TPC was estimated for Xiao Tainang at 97.47 mg GAE/100 g FW, followed by Aozhou, Da Tainang and Shuixian cultivars, while Lvsong showed the lowest content. The measured levels of total phenolic content in the pulp samples were in agreement, as reported earlier in different varieties of mango, such as Tommy Atkins and Pica mango from the USA and Chile [24,25,26,27], as well as in Brazil and Ecuador [37] and the Haden variety from Mexico [39].

**Table 2:** Total phenolics, flavonoids and anthocyanins contents of mango pulp and peel.

Varieties	Total Phenolics Content		Total Flavonoids Content		Total Anthocyanins Content	
	Pulp	Peel	Pulp	Peel	Pulp	Peel
F1	22.06 ^g^ ± 0.27	462.2 ^h^ ± 10.06	3.069 ^fg^ ± 0.21	34.61 ^d^ ± 1.29	nd	0.006 ^b^ ± 0.01
F2	62.45 ^d^ ± 1.25	622.4 ^g^ ± 4.46	8.321 ^b^ ± 0.15	48.87 ^c^ ± 1.50	nd	nd
F3	48.77 ^e^ ± 0.34	997.9 ^e^ ± 19.61	2.995 ^fg^ ± 0.15	29.85 ^e^ ± 1.18	0.0005 ^a^ ± 0.0	0.659 ^a^ ± 0.01
F4	74.41 ^c^ ± 3.00	2805 ^b^ ± 17.42	4.578 ^d^ ± 0.15	75.35 ^a^ ± 2.68	nd	0.049 ^b^ ± 0.01
F5	28.14 ^f^ ± 0.91	927.2 ^f^ ± 17.07	0.904 ^i^ ± 0.07	19.91 ^f^ ± 0.70	nd	nd
F6	83.49 ^b^ ± 2.07	1131 ^d^ ± 12.87	9.252 ^a^ ± 0.18	59.31 ^b^ ± 3.71	0.0001 ^a^ ± 0.0	0.647 ^a^ ± 0.07
F7	45.78 ^e^ ± 0.67	1376 ^c^ ± 15.22	2.583 ^h^ ± 0.54	19.91 ^f^ ± 0.59	0.0004 ^a^ ± 0.0	nd
F8	97.47 ^a^ ± 6.76	4071 ^a^ ± 17.47	5.735 ^c^ ± 0.45	59.20 ^b^ ± 1.89	nd	0.014 ^b^ ± 0.03
F9	51.68 ^e^ ± 0.66	1145 ^d^ ± 15.07	3.500 ^e^ ± 0.20	27.49 ^e^ ± 0.88	nd	0.015 ^b^ ± 0.02

Phenolic content expressed as mg of gallic acid equivalents per 100 g of fresh weight (FW); flavonoid content expressed as mg of catechin equivalents per 100 g of FW; anthocyanin content expressed as mg/100 mL of cyanidin 3-glucoside equivalents on a fresh weight basis; nd, not detected; different letters (a–i) within the columns indicate significant difference at *p* < 0.05; values are the means of three replicates ± SD.

### 2.3. Total Flavonoid Content

Estimated values of total flavonoid content (TFC) in the studied samples are given in Table 2, which revealed that peel samples exhibited a significant level of TFC compared to the pulp samples. These findings were in agreement that in mango, the peel contains more flavonoids than pulp [15]. In the peel samples, TFC ranged between 75.35–19.90 mg catechin equivalent (CE)/100 g FW. The peel of the Da Tainang cultivar showed the highest content of total flavonoids at 75.35 mg CE/100 g FW, whereas the lowest values were calculated in the Kaite and Xiangya cultivars. These concentrations were statistically different (*p* < 0.05) among the studied samples. In the case of pulp samples, the concentration of total flavonoids was maximum in Aozhou (9.252 mg CE/100 g FW), whereas the minimum content was estimated for the Kaite variety at 0.904 mg CE/100 g FW. Our results demonstrated that the measured levels of total flavonoid content were comparatively higher than reported previous reports for the pulp and peel of Pica variety from Chile [24], for the pulp of Mallika variety from China [25] and Ataulfo mango [15,40].

### 2.4. Total Anthocyanin Contents

Anthocyanins are well known because of their antioxidant properties and their pigmenting power that make them attractive to be used as food colorants [1,32]. It has been reported that anthocyanins are comparatively higher in ripe mango peel than raw peel [32]. Like phenolic and flavonoid contents, total anthocyanin content (TAC) was also estimated to be more in the peel samples than pulp (Table 2). In the peel samples, the maximum contents of total anthocyanins were determined in the Guifei and Aozhou cultivars at 0.659 and 0.647 mg/100 mL of cyanidin 3-glucoside equivalents on a fresh weight basis, respectively. However, the contents of total anthocyanins in the pulp samples were very low. Anthocyanin contents were below the detection limit in the peel of Shuixian, Kaite and Xiangya and in the pulp of Lvsong, Shuixian, Da Tainang, Kaite, Xiao Tainang and Jidan. Compared to the literature, the present findings indicated that anthocyanin contents in Chinese cultivars were comparatively lower than reported in the peel of Indian mango [32], which might be attributed to genetic variation and their origin. Likewise, the reported level of TAC (2.1–26.8 mg of cyanidin 3-glucoside equivalent/100 g) in the peel of apple [41] was significantly higher than mango peel. In general, the present analysis revealed that mango peel contains more contents of total phenolics, flavonoids and anthocyanins. Therefore, it could be an excellent source of natural antioxidants and bioactive ingredients of functional food.

### 2.5. Phenolic Acids Composition

**Figure 1:**
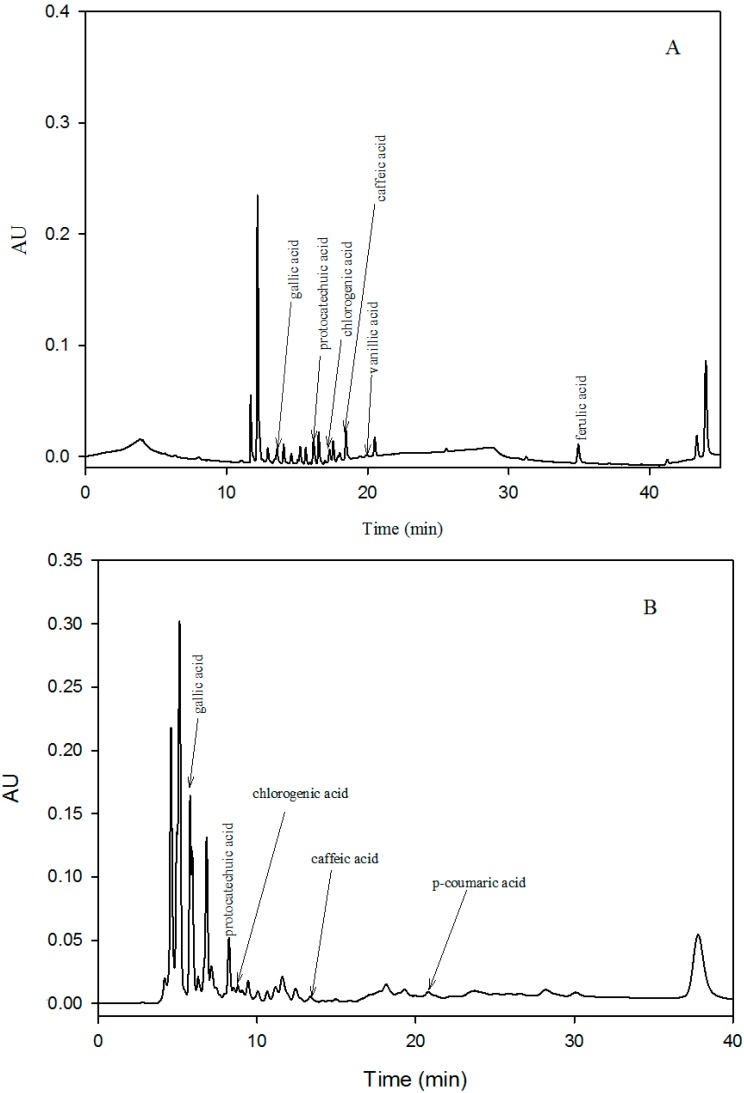
(**A**) HPLC-chromatogram showing the phenolic acid composition in the pulp samples of mango recorded at 280 and 320 nm; and (**B**) HPLC-chromatogram showing the phenolic acid composition in the peel samples of mango recorded at 280 and 320 nm.

Measured levels of gallic acid, caffeic acid, chlorogenic acid, protocatechuic acid, vanillic acid and *p*-coumaric acid, which were identified and quantified for the first time by the HPLC method in the pulp and peel of different mango cultivars grow in China, are presented in Table 3 and Table 4. Comparatively, elevated levels of phenolic acids were determined in the peel samples. Though similar types of phenolic acids have been reported before in different varieties of mango [10,15,36], in the case of pulp, concentrations of phenolic acids were different than reported for Ataulfo [15,40], Kent [14] and Tommy Atkins [34] varieties of mango. Gallic acid, caffeic acid, chlorogenic acid and protocatechuic acid were identified in all studied samples (Figure 1A,B). Ferulic and vanillic acids were determined only in the pulp samples (Figure 1A), while *p*-coumaric acid was estimated in the peel samples only (Figure 1B). In the pulp samples, ferulic acid was predominant, followed by protocatechuic, chlorogenic, gallic, vanillic and caffeic acids. However, in the peel samples, gallic acid was predominant, followed by protocatechuic acid, chlorogenic acid, caffeic acid and *p*-coumaric acid. The Xiao Tainang cultivar exhibited the highest content of ferulic acid (33.75 mg/100 g) on a fresh weight basis, followed by Aozhou and Guifei. The lowest concentration of ferulic acid was present in the pulp of Lvsong, whereas in the Da Tainang and Kaite, cultivars ferulic acid contents were below the detection limit.

In the peel samples, the gallic acid concentration varied from 79.15–1.450 mg/100 g FW. Gallic acid was maximum in the peel of the Da Tainang cultivar, whereas the minimum content was determined in Aozhou. In the pulp samples, the highest content of gallic acid was estimated for Aozhou, followed by the Lvsong and Da Tainang cultivars at 2.982, 2.492 and 2.369 mg/100 g FW, respectively. These values were significantly different at *p* < 0.05. In the peel samples, measured values of gallic acid were compatible with the reported levels in the Ataulfo variety, whereas pulp contained a lower concentration than the reported levels in previous studies [15]. It was noted that, in the peel of the Jidan cultivar, chlorogenic acid was maximum at 27.19 mg/100 g FW, whereas the lowest value was estimated in the peel of Shuixian. However, in the pulp samples, chlorogenic acid ranged from 0.957 mg/100 g FW in Xiangya to 6.147 mg/100 g FW in the Aozhou cultivar, and these values were considerably lower compared to Ataulfo mango [15].

A significant level of protocatechuic acid was quantified in the peel of Xiao Tainang at 64.33 mg/100 g FW, followed by the Aozhou and Jidan cultivars with a significant difference at *p* < 0.05. In the case of pulp samples, the maximum concentration of protocatechuic acid was estimated in the pulp of Aozhou, followed by the Jidan and Xiao Tainang cultivars. In the pulp samples, caffeic acid ranged between 1.117 and 0.250 mg/100 g FW. The highest concentration of caffeic acid was determined in the peel of Jidan at 13.62 mg/100 g FW, followed by Xiangya and Xiao Tainang at 7.070 and 2.989 mg/100 g FW, respectively, whereas it was undetectable in the peel of other cultivars.

**Table 3:** Phenolic acids content (mg/100 g FW) in the pulp samples of mango cultivars.

Varieties	Gallic Acid	Caffeic Acid	Protocatechuic Acid	Chlorogenic Acid	Vanillic Acid	Ferulic Acid
F1	2.492 ^ab^ ± 0.11	0.562 ^a^ ± 0.03	1.234 ^cde^ ± 0.084	1.589 ^c^ ± 0.148	0.642 ^de^ ± 0.032	1.206 ^e^ ± 0.21
F2	1.543 ^bc^ ± 0.15	0.264 ^a^ ± 0.04	1.116 ^de^ ± 0.073	3.779 ^b^ ± 0.167	1.042 ^b^ ± 0.069	20.31 ^bc^ ± 1.15
F3	2.359 ^ab^ ± 0.08	0.485 ^a^ ± 0.10	0.767 ^e^ ± 0.011	1.298 ^cd^ ± 0.051	0.669 ^de^ ± 0.125	28.69 ^ab^ ± 2.14
F4	2.369 ^ab^ ± 0.41	0.707 ^a^ ± 0.15	1.768 ^bcd^ ± 0.033	1.040 ^d^ ± 0.065	0.900 ^bc^ ± 0.079	nd
F5	1.788 ^bc^ ± 1.02	0.767 ^a^ ± 0.33	0.984 ^de^ ± 0.042	0.971 ^d^ ± 0.021	0.802 ^cd^ ± 0.026	nd
F6	2.982 ^a^ ± 0.23	1.117 ^a^ ± 0.10	6.826 ^a^ ± 0.532	6.147 ^a^ ± 0.407	1.625 ^a^ ± 0.095	28.96 ^ab^ ± 2.83
F7	0.927 ^c^ ± 0.08	0.250 ^a^ ± 0.04	1.211 ^cde^ ± 0.078	0.957 ^d^ ± 0.061	0.565 ^d^ ± 0.090	7.207 ^de^ ± 3.47
F8	2.168 ^ab^ ± 0.25	0.894 ^a^ ± 0.09	2.046 ^bc^ ± 0.044	1.246 ^cd^ ± 0.103	1.461 ^a^ ± 0.101	33.75 ^a^ ± 1.44
F9	2.112 ^ab^ ± 0.06	0.481 ^a^ ± 0.04	2.288 ^b^ ± 0.116	1.335 ^cd^ ± 0.035	0.942 ^bc^ ± 0.017	15.48 ^cd^ ± 5.97

Different letters (a–e) within the columns indicate significant difference at *p* < 0.05; values are the means of three replicates ± SD; nd.: not detected.

**Table 4:** Phenolic acids content (mg/100 g FW) in the peel samples of mango cultivars.

Varieties	Gallic Acid	Caffeic Acid	Protocatechuic Acid	Chlorogenic Acid	*p*-Coumaric Acid
P1	7.376 ^b^± 1.01	nd	8.396 ^b^± 1.57	4.523 ^a^± 0.77	nd
P2	2.710 ^b^± 2.35	nd	3.167 ^b^± 0.25	4.405 ^a^ ± 0.08	nd
P3	21.38 ^b^± 1.15	nd	3.989 ^b^± 0.32	4.462 ^a^ ± 0.30	nd
P4	79.15 ^a^ ± 8.61	nd	7.807 ^b^± 1.63	9.409 ^a^ ± 1.16	0.291 ^a^ ± 0.50
P5	16.57 ^b^ ± 3.82	nd	3.077 ^b^± 0.51	5.944 ^a^ ± 0.04	nd
P6	1.450 ^b^ ± 1.27	nd	35.23 ^ab^± 9.10	19.65 ^a^ ± 1.50	nd
P7	10.83 ^b^ ±2.42	3.303 ^b^ ± 0.683	2.974 ^b^± 0.20	25.37 ^a^ ± 2.70	nd
P8	6.672 ^b^ ±1.78	4.484 ^b^± 0.105	64.33 ^a^± 14.4	21.96 ^a^ ± 2.14	0.676 ^a^ ± 0.61
P9	1.834 ^b^ ± 1.59	14.43 ^a^ ± 2.97	12.63 ^b^± 2.18	27.19 ^a^ ± 3.02	nd

Different letters (a,b) within the columns indicate significant difference at *p* < 0.05; values are the means of three replicates ± SD; nd.: not detected.

### 2.6. Antioxidant Capacity

**Figure 2:**
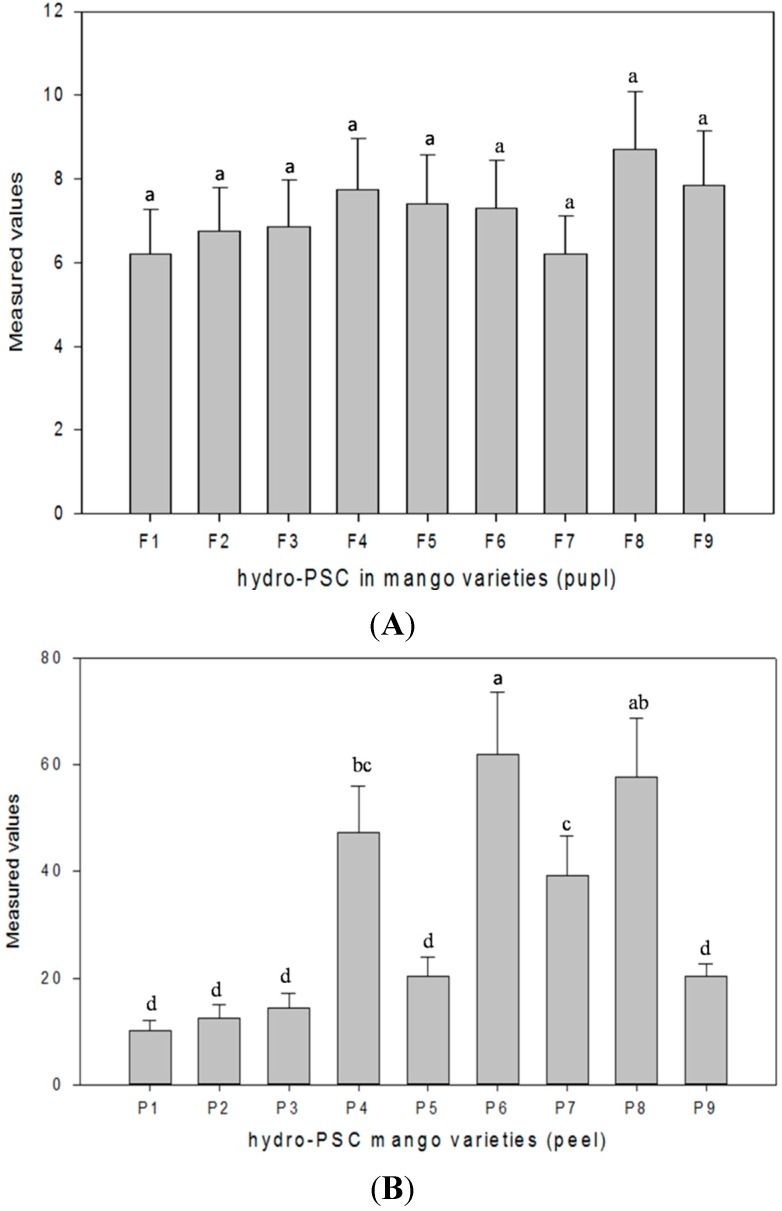
(**A**) Hydro-PSC values (μM vitamin C equivalent/g FW) in pulp samples; the means of three replicates ± SD; letters "a" indicate significant difference at *p* < 0.05; (**B**) Hydro-PSC values (μM vitamin C equivalent/g FW) in peel samples, the means of three replicates ± SD; different letters “a–d” indicate significant difference at *p* < 0.05.

**Figure 3:**
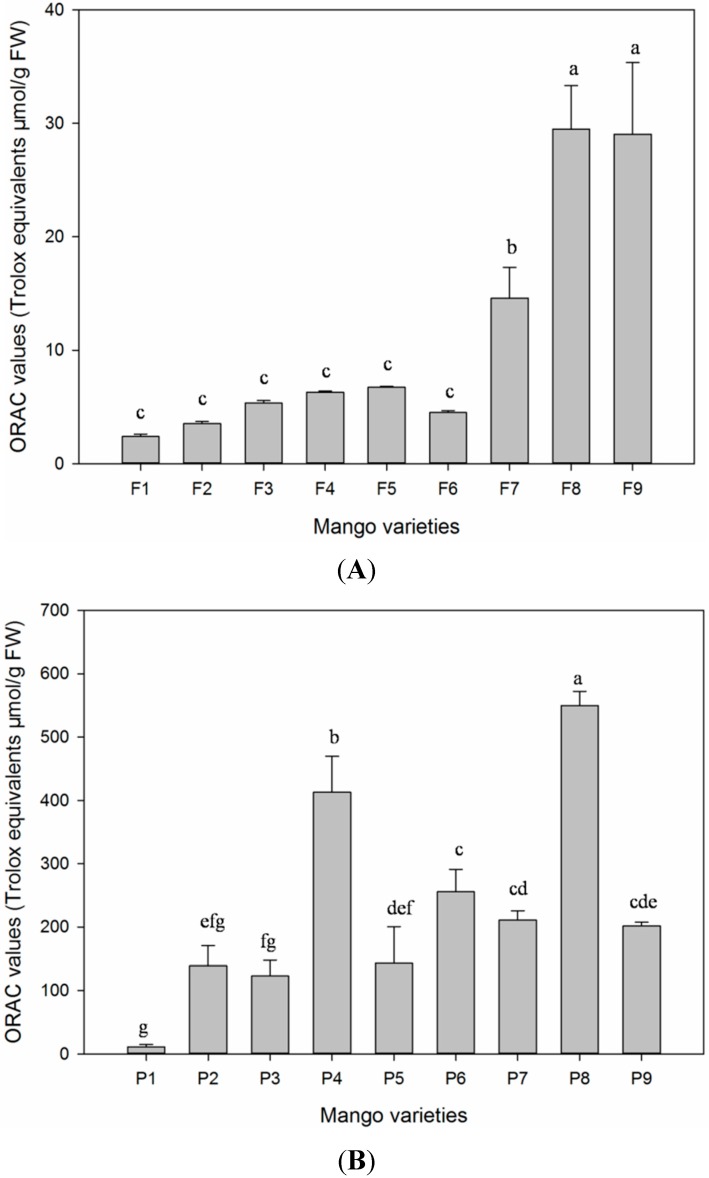
(**A**) ORAC value in pulp samples, the means of three replicates ± SD; different letters “a–c” indicate significant difference at *p* < 0.05; (**B**) ORAC value in peel samples; the means of three replicates ± SD; different letters “a–g” indicate significant difference at *p* < 0.05.

The results of *in vitro* antioxidant capacity determined PSC and ORAC assays are presented in Figure 2 and Figure 3. In general, peel samples showed more antioxidant capacity compared to pulp. The hydro-PSC method was used for the first time to evaluate the antioxidant capacity of mango. In the peel samples, the PSC values ranged between 61.91 and 10.25 μM vitamin C equivalent/g FW. The highest peroxyl radical scavenging capacity was shown by the Aozhou cultivar, followed by Xiao Tainang and Da Tainang, whereas Lvsong showed the lowest level (Figure 2B). In the pulp samples, the maximum PSC value was calculated for the Xiao Tainang cultivar at 8.713 μM vitamin C equivalent/g FW (Figure 2A).

The results of oxygen radical absorbance capacity (ORAC) indicated that peel and pulp of the Xiao Tainang cultivar showed antioxidant capacity determined by the ORAC assay at 549.8 and 29.50 μM Trolox equivalent/g FW (Figure 3A,B) among all of the studied samples. In the previous studies, the antioxidant activity of mango has been estimated by different methods [15,24,25,26,27,32], which are incomparable to our findings. However, it was noted that measured values of oxygen radical absorbance capacity in mango pulp were compatible to [25,27]. To our knowledge, mango peel has never been analyzed to determine the oxygen radical absorbance capacity before.

**Table 5:** Correlation coefficient matrix between phenolics content and antioxidant properties in mango pulp.

	TPC	TFC	TAC	GA	CA	PA	CIA	VA	FA	PSC	ORAC
TPC	1.000										
TFC	0.714 *	1.000									
TAC	−0.964	−0.968	1.000								
GA	0.287	0.349	−0.586	1.000							
CA	0.485	0.310	−0.895	0.735 *	1.000						
PA	0.520	0.661	−0.994	0.577	0.694 *	1.000					
ClA	0.390	0.858 **	−0.967	0.420	0.391	0.807 **	1.000				
VA	0.822 **	0.784 *	−0.959	0.499	0.736 *	0.766 *	0.676 *	1.000			
FA	0.663	0.591	−0.330	0.287	0.273	0.402	0.453	0.653	1.000		
PSC	0.675 *	0.172	−0.659	0.313	0.580	0.210	−0.088	0.635	0.378	1.000	
ORAC	0.368	−0.109	0.383	−0.177	0.038	−0.013	−0.352	0.253	0.319	0.648	1.000

TPC, Total phenolic content; TFC, Total flavonoid content; TAC, Total anthocyanin content; GA, Gallic acid; CA, Caffeic acid; PCA, Protocatechuic acid; ClA, Chlorogenic acid; VA, Vanillic acid; FA, Ferulic acid; PSC, Peroxyl radical scavenging capacity; ORAC, Oxygen radical absorbance capacity; * Correlation is significant at the 0.05 level (2-tailed); ** Correlation is significant at the 0.01 level (2-tailed).

**Table 6:** Correlation coefficient matrix between phenolics content and antioxidant properties in mango peel.

	TPC	TFC	TAC	GA	CA	PCA	ClA	*p*CA	PSC	ORAC
TPC	1.000									
TFC	0.589	1.000								
TAC	−0.380	−0.095	1.000							
GA	0.379	0.492	−0.168	1.000						
CA	0.042	−0.387	−0.405	−0.287	1.000					
PCA	0.723 *	0.491	−0.097	−0.237	0.016	1.000				
ClA	0.372	−0.041	−0.211	−0.282	0.786 *	0.463	1.000			
*p*CA	0.962 **	0.585	−0.441	0.254	−0.059	0.782 *	0.241	1.000		
PSC	0.702 *	0.615	0.106	0.192	−0.025	0.713 *	0.564	0.587	1.000	
ORAC	0.977 **	0.655	−0.332	0.356	0.048	0.753 *	0.441	0.920 **	0.805 **	1.000

TPC, Total phenolic content; TFA, Total flavonoid content; TAC, Total anthocyanin content; GA, Gallic acid; CA, Caffeic acid; PCA, Protocatechuic acid; ClA, Chlorogenic acid; *p*CA, *p*-Coumaric acid; PSC, Peroxyl radical scavenging capacity; ORAC, Oxygen radical absorbance capacity; * Correlation is significant at the 0.05 level (2-tailed); ** Correlation is significant at the 0.01 level (2-tailed).

### 2.7. Correlations

In view of the fact that a large number of different antioxidants contribute to the total antioxidant capacity, it is not yet clear which components are more accountable for the observed antioxidant capacity [26]. Significant correlations between phenolic compounds and antioxidant activity in various kinds of fruits have been reported in previous studies [2,42,43,44]. Table 5 and Table 6 showed correlation coefficient matrices between phenolic content (*i.e.*, TPC, TFC, TAC and phenolic acids) and antioxidant capacity in the pulp and peel samples. In the peel samples, highly significant coefficients of determination were calculated between TPC-ORAC, TPC-*p*-coumaric acid, ORAC-*p*-coumaric acid and ORAC-PSC (0.977, 0.962, 0.920 and 0.805, respectively). In the pulp samples, significant correlations were noted between TFC and chlorogenic acid (*r* = 0.858), TPC and vanillic acid (*r* = 0.822), protocatechuic acid-and chlorogenic acid (*r* = 0.807) and TFC-vanillic acid (*r* = 0.784), while total phenolic content exhibited a strong relationship with hydro-PSC (*r* = 0.675). These results were consistent with previous studies [45] and indicated that phenolic compounds contribute significantly to the antioxidant capacity. An inverse relationship between the consumption of foods rich in phenolic acids, such as chlorogenic acid and gallic acid, and the occurrence of different diseases has been suggested previously [25]. However, we observed significant correlations between phenolic acids and antioxidant activity in mango cultivars. Our data revealed that mango peel in particular is an excellent source of phenolic compounds, which are major source of natural antioxidants [25]. These findings are in agreement with the declaration that the antioxidant capacity of fruits and vegetables appears to be largely influenced by non-vitamin C phytochemicals [46].

## 3. Materials and Methods

### 3.1. Chemicals and Material

Ascorbic acid (ASA), aluminum chloride, chloranil, tetrahydrofuran (THF), catechin hydrate, vanillin, Folin-Ciocalteu reagent, dichlorofluorescein diacetate (DCFH-DA), 2,2ʹ-azobis-amidinopropane (ABAP), gallic acid and 6-hydroxy-2,5,7,8-tetramethylchroman-2-carboxylic acid (Trolox) were purchased from Sigma Chemical Co. (St. Louis, MO, USA). Potassium dihydrogen phosphate (KH_2_PO_4_), sodium borohydride (NaBH_4_), ethanol, acetone, acetic acid, hydrochloric acid (HCl), di-potassium hydrogen phosphate (K_2_HPO_4_), sodium carbonate, sodium bicarbonate (NaHCO_3_) and acetonitrile were purchased from Aladdin Co. (Shanghai, China). Protocatechuic acid, chlorogenic acid, (+)-catechin, caffeic acid, *p*-coumaric acid, ferulic acid and formic acid of HPLC grade were purchased from Sigma-Aldrich, Inc. (St. Louis, MO, USA). Methanol and acetonitrile of HPLC grade were purchased from Anpel Scientific instrument Co., Ltd. (Shanghai, China).

**Figure 4:**
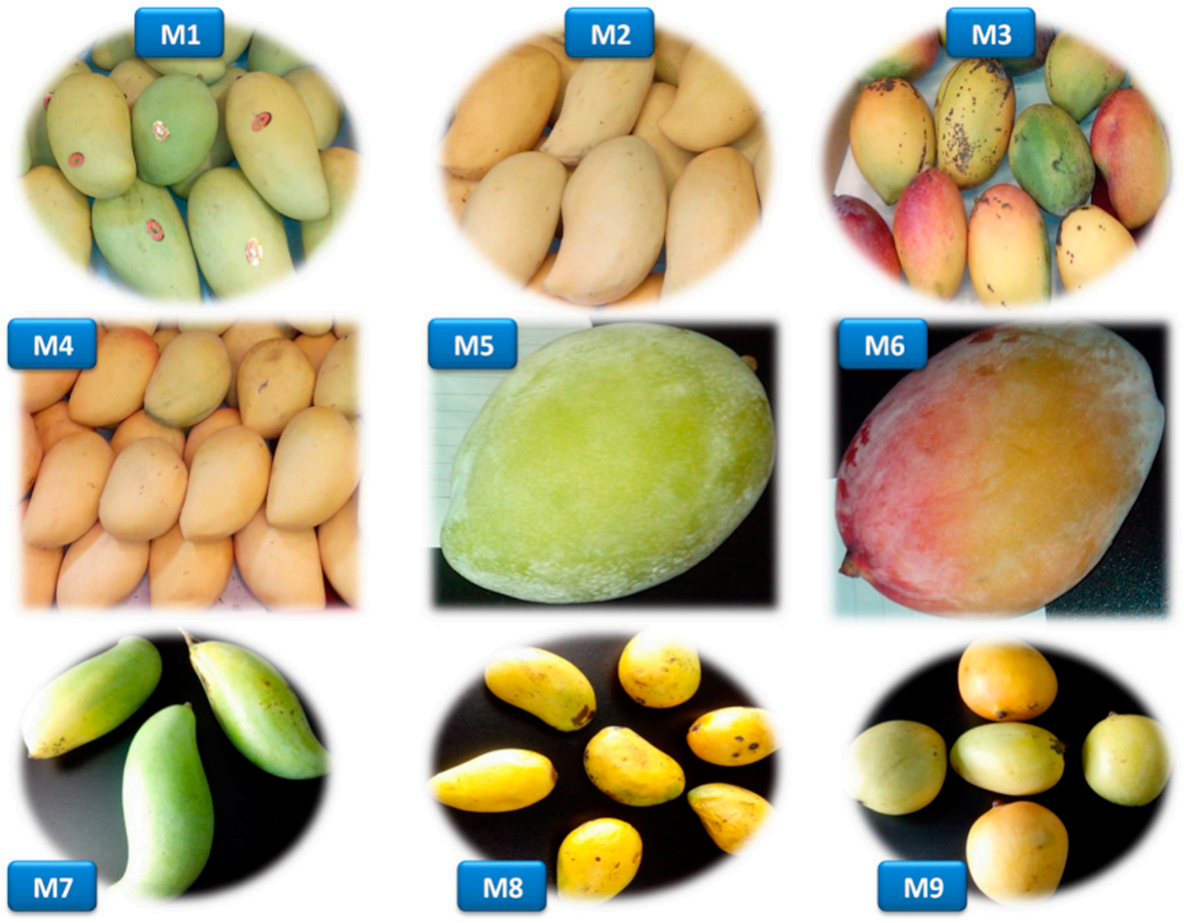
Mango cultivars used in the present analysis. M1, Luzon mango (Lvsong); M2, Narcissus mango (Shuixian); M3, Royal mango (Guifei); M4, Big Tainong mango (Da Tainang); M5, Keitt mango (Kaite); M6, Australian mango (Aozhou); M7, Thai mango (Xiangya); M8, Small Tainong mango (Xiao Tainang); M9, Egg mango (Jidan).

Fresh and fully-ripened mango fruits of nine different cultivars, including Luzon (Lvsong), Narcissus (Shuixian), Royal (Guifei), Big Tainong (Da Tainong), Keitt (Kaite), Australian mango (Aozhou), Thai mango (Xiangya), Small Tainong (Xiao Tainong) and Egg mango (Jidan), were purchased from the supermarkets of Guangzhou city in Guangdong province of China and transported instantly to the laboratory for analysis (Figure 4). All fruits were properly cleaned with de-ionized water and stored at −40 °C until analysis.

### 3.2. Moisture Content

The moisture content in the pulp and peel was determined by a modified oven-dried method [46]. Briefly, 10 g of pulp and 5 g of peel samples were dried in an oven at 105 °C until constant weight. Each drying test was performed in triplicate, and data were presented as the mean ± standard deviation (SD) of triplicates.

### 3.3. Extraction

Phenolics were extracted following the method as described by [47], with modifications [48]. Briefly, 5 g of fresh pulp and 3 g of fresh peel in triplicate were blended five times each with 25 mL of 80% chilled acetone for 5 min, followed by homogenization for 5 min in an electric homogenizer. Homogenates were centrifuged at 2500 rpm for 10 min; supernatants were pooled in rotary flasks and evaporated using a rotary evaporator at 45 °C until 10% of the filtrate was left behind. The filtrates were reconstituted with water to a final volume of 10 mL and stored at −40 °C for further analysis.

### 3.4. Determination of Total Phenolic Content

The Folin-Ciocalteu colorimetric method, as described earlier [49], with modifications [50], was used to determine the total phenolic content in the peel and pulp samples. All extracts were diluted with Milli-Q water to get readings falling within the range of the standard curve concentration: 0.0–600.0 µg gallic acid/mL. One hundred microliters of gallic acid solution or extracts were added to 0.4 mL of Milli-Q water in each test tube, followed by the addition of Folin-Ciocalteu reagent (0.1 mL). The solutions were allowed to react for 6 min to ensure the complete and speedy reaction of the Folin-Ciocalteu reagent with oxidizable phenolates in the sample. Then, 1 mL of 7% sodium carbonate solution was added to neutralize the mixture, followed by the addition of 0.8 mL Milli-Q water to adjust the final volume to 2.4 mL. The samples were mixed and allowed to stand for 90 min at room temperature. After color development, absorbance was measured at 760 nm on a DU 730 Nucleic Acid/Protein analyzer (BECKMAN, Inc., Fullerton, CA, USA). Total phenolic contents were calculated based on the standard curve of known gallic acid concentrations, and final values were expressed as milligrams of gallic acid equivalent per 100 grams on a fresh weight basis (mg GAE/100 g FW). Data were presented as the mean ± SD for triplicates analyses.

### 3.5. Estimation of Total Flavonoid Content

Total flavonoid content was estimated by the sodium borohydride/chloranil method (SBCM) as established in our laboratory [30]. Briefly, 1 mL of each extract was added into test tubes (15 × 150 mm), then kept under nitrogen gas until dried and reconstituted with 1 mL of tetrahydrofuran/ethanol (THF/EtOH, 1:1, *v*/*v*). Freshly-prepared catechin hydrate (0.3–10.0 mM) in 1 mL of THF/EtOH (1:1, *v*/*v*) was used as the standard for analysis. Zero-point-five milliliters of each (NaBH_4_ (50 mM) and AlCl_3_ (74.6 mM)) solution were added into all test tubes with samples or standards and shaken on an orbital shaker at room temperature for 30 min. Additionally, 0.5 mL of NaBH_4_ (50.0 mM) solution were added into each test tube and shaken for another 30 min under the same condition. After shaking, 2.0 mL of chilled acetic acid (0.8 M) were thoroughly mixed, and the mixture was kept in the dark for 15 min. Then, 1 mL chloranil solution (20.0 mM) was added in each tube, and the mixture was heated at 95 °C in a shaking bath for 60 min. The reaction solutions were cooled with tap water, and the final volume was kept at 4 mL using methanol. One milliliter of 16% vanillin solution (*w*/*v*) was added into each tube, followed by the addition of 2 mL HCl (12 M), then mixed thoroughly and kept in the dark for 15 min. The reaction solutions were centrifuged at 2500 rpm for 10 min, and absorbance was immediately measured at 490 nm against a blank using a DU 730 Nucleic Acid/Protein analyzer (BECKMAN, Inc.). Total flavonoid content in each sample was calculated, using the standard curve of catechin hydrate concentration. The final value was expressed as milligrams of catechin equivalent per 100 gram of fresh weight (mg CE/100 g FW), and data were reported as the mean ± SD for triplicate analyses.

### 3.6. Determination of Total Anthocyanin Content

Total anthocyanin content was determined following the method as explained by [41]. Acetone extracts of pulp and peel samples in triplicate were mixed carefully with 0.025 M potassium chloride buffer (pH = 1) in 1:6 ratio. The absorbance was measured at 515 and 700 nm against distilled water blank (BECKMAN). Afterword, the extracts were mixed with sodium acetate buffer (pH = 4.5); absorbance was measured at the same wavelengths, and the total content of anthocyanins was calculated using the formula as follows:

$$\text{Total anthocyanins}\;(\text{mg}/100\;\text{g of FW of samples})=\;A\times \;M_{W}\;\times \;\text{1000}/(\varepsilon \;\times C)$$

where *A* is absorbance = (*A*515 − *A*700) pH 1.0 − (*A*515 − *A*700) pH 4.5; *M*_W_ is the molecular weight for cyanidin 3-glucoside = 449.2; ε is the molar absorptivity of cyanidin 3-glucoside = 26,900; and *C* is the concentration of the buffer in mg/mL. Anthocyanin content was expressed as milligrams of cyanidin 3-glucoside equivalent per 100 g on fresh weight basis (mg CGE/100 g FW), and data were reported as the mean ± SD for triplicates analyses.

### 3.7. Identification and Quantification of Phenolic Acids

Phenolic acids in the pulp and peel extracts of mango cultivars were determined by the method explained by [15]. Samples were injected automatically into an HPLC system (Waters Corp., Milford, MA, USA) equipped with a photodiode array detector. Absorption spectra for the main peaks were recorded at 280 and 320 nm. The HPLC system was equipped with a C18 reverse phase column (250 mm × 4.6 mm, 5 μm);the mobile phase was composed of 1% formic acid (A) and acetonitrile (B), and the isocratic elution gradient was 20% (B) in 40 min at a flow rate of 0.6 mL/min at 25 °C. The injection volume of the sample was 20 µL. Peaks were identified on the basis of retention time and chromatographs of the standards. Phenolic acids were identified and quantified on the basis of calibration curves and were expressed as mg phenolics per 100 g of FW. Data were reported as the mean ± SD for triplicate analyses.

### 3.8. Antioxidant Capacity Assays

Currently, researchers are paying more attention to natural antioxidants present in fruits, vegetables and whole foods because of their safety and potential nutritional and therapeutic effects [51]. The antioxidant potential of commonly-consumed tropical and subtropical fruit has been rated in the order of guava > mango > papaya > lemon [52]. Owing to the complex reactivity of phytochemicals, the antioxidant capacity of food and food extracts cannot be estimated by only a single method. However, at least two test systems have been recommended to establish legitimacy [53]. Consequently, the antioxidant capacity in the peel and pulp samples of mango cultivars was evaluated by the peroxyl scavenging capacity (PSC) and oxygen radical antioxidant capacity (ORAC) methods.

#### 3.8.1. Hydrophilic Peroxyl Radical Scavenging Capacity Assay

The peroxyl scavenging capacity (PSC) assay is based on the oxidation of DCFH by peroxyl radicals and is used to determine the antioxidant capacity in hydrophilic and lipophilic extracts of fruits, vegetables, grains and whole food [47].The hydrophilic peroxyl radical scavenging capacity (hydro-PSC) assay, as explained by [47], with modifications [48,54], was used to assess antioxidant capacity in the pulp and peel of mango cultivars. Seventy five millimolar phosphate buffer (pH 7.4) was used to dilute samples in appropriate concentrations. Ascorbic acid and gallic acid were made fresh and diluted to (6.3, 4.8, 3.2, 2.4, 1.0) and (5, 3.5, 2.7, 1.4, 0.9) μg/mL concentrations, respectively, using phosphate buffer (75 mM, pH 7.4). The reaction mixture contained phosphate buffer (75 mM, pH 7.4), ABAP (40 mM), DCFH dye (13.26 μM) and the suitable amount of the pure antioxidant compound or sample extract. The dye was prehydrolyzed with 1 mM KOH to eradicate di-acetate before use and the reaction was carried out at 37 °C, in a total volume of 250 µL using a 96-well plate. Fluorescence generation was observed (excitation at 485 nm and emission at 538 nm) on a Fluoroskan Ascent fluorescent spectrophotometer (SoftMax systems, Molecular Devices, Sunnyvale, CA, USA). Data were analyzed using SoftMax Pro Software, Version 6.2 (SoftMax systems, Molecular Devices) running on a PC. The areas under the fluorescence reaction time kinetic curve (AUC) for both control and samples were included and used as the basis for the determination of peroxyl radical scavenging capacity (PSC) using equation:

PSC(Value)=1−(SA/CA)

where *SA* is the AUC for the sample or standard dilution and *CA* is the AUC for the control reaction. Compounds or extracts inhibiting the oxidation of DCFH produced lesser *SA* and higher *PSC* values. EC_50_, the dose requisite to cause 50% inhibition (*PSC* unit = 0.5) for each pure compound or sample extract, was used to assess antioxidant activity of different compounds or samples. Final values of hydro-PSC were expressed as μmol of vitamin C equivalent per 100 g of FW (μM vCe/100 g of FW), and data were reported as the mean ± SD of each triplicate.

#### 3.8.2. Oxygen Radical Scavenging Capacity Assay

The ORAC assay is a widely-used method to analyze the oxygen radical absorbance capacity of plant species extracts. This assay is based on free radical damage to a fluorescent probe through a change in its fluorescence intensity [55]. In the typical ORAC assay, the fluorescent loss of probes as phycoerythrin or fluorescein is followed over time in the absence and presence of antioxidant [41]. The oxygen radical absorbance capacity (ORAC) assay, as described by [55], with modifications [56], was conducted to measure the total antioxidant activity of the studied samples. Briefly, 20 μL of sample extracts in triplicate, diluted with 75 mM phosphate buffer (pH 7.4), were added in 96-well microplate, followed by the addition of 200 μL of fluorescein (0.96 μM), and incubated at 37 °C for 20 min. Outer wells were kept empty to avoid variation from inner wells. After incubation, 20 μL of freshly-prepared 119.4 mM AAPH in 75 mM phosphate buffer (pH 7.4) were added into each well, and the fluorescence intensity was measured immediately for 35 cycles every 4.5 min at an excitation of 485 nm and emission of 535 nm by the FilterMax F5 Multi-Mode Microplate Reader (Molecular Devices, Sunnyvale, CA, USA). Different concentrations of Trolox (range 6.25–50 μM) were used as a control. ORAC values were calculated by extrapolation on a calibration curve and expressed as the mean ± SD micromoles of Trolox equivalent per 100 g of fresh weigh (μM TE/100 g of FW) for three replicates.

### 3.9. Statistical Analysis

Statistical analyses were performed using SPSS software 13.0 (SPSS Inc., Chicago, IL, USA), and the dose effect was analyzed using Calcusyn software Version 2.0 (Biosoft, Cambridge, UK). Results were subjected to ANOVA, and differences among means were located using Tukey’s multiple comparison test. A *p*-value less than 0.05 (*p* < 0.05) was regarded as statistically significant. Basic statistical parameters and correlation coefficients among the measured variables were also calculated. All data were reported as the mean ± SD for three replicates.

## 4. Conclusions

The present study was focused on comparative assessment of phenolic content and *in vitro* antioxidant capacity in the pulp and peel of mango cultivars. Though phenolic compounds showed a significant contribution in the inhibition of free radicals, the antioxidant capacity of mango and other fruits is not only due to the content of phenolic acids. It may also be due to the presence of various bioactive compounds, such as carotenoids, vitamins and other polyphenolics phytochemicals present in the pulp and peel of mango, which were not identified in the present study. Our results showed that the Xiao Tainang and Aozhou cultivars contained maximum phenolic content and exhibited remarkable antioxidant capacity. Gallic acid and protocatechuic acid were predominant in the peel and pulp of the studied samples. Highly significant correlations (*r* = 0.997, 0.962, 0.922, *etc.*) were calculated between phenolic and antioxidant properties, particularly in the peel samples. The present study revealed that the antioxidant capacity of mango peel is due to the synergistic actions of phenolics and other bioactive compounds present in it. Therefore, it is suggest that mango peel may contribute to promoting human health as a functional food or a value-added ingredient. To our knowledge, this is the first report on the comparative assessment of phenolic compounds and antioxidant capacity determined by ORAC and hydro-PSC assays in the peel and pulp of mango cultivars in China, particularly in peel. However, additional studies are desirable to assess the bio-absorption, mechanism of action and associations between these compounds after consumption.
